# Insights into the impact and use of research results in a residential long-term care facility: a case study

**DOI:** 10.1186/1748-5908-7-90

**Published:** 2012-09-13

**Authors:** Lisa A Cranley, Judy M Birdsell, Peter G Norton, Debra G Morgan, Carole A Estabrooks

**Affiliations:** 1 Faculty of Nursing, Edmonton Clinic Health Academy, University of Alberta, 11405 87 Avenue, Edmonton, AB, Canada; 2 On Management Health Group, 1700 Varsity Estates Drive, Calgary, AB, Canada; 3 Faculty of Medicine, University of Calgary, 3330 Hospital Drive, Calgary, AB, Canada; 4 Canadian Centre for Health and Safety in Agriculture, University of Saskatchewan, 103 Hospital Drive, Saskatoon, SK, Canada

## Abstract

**Background:**

Engaging end-users of research in the process of disseminating findings may increase the relevance of findings and their impact for users. We report findings from a case study that explored how involvement with the Translating Research in Elder Care (TREC) study influenced management and staff at one of 36 TREC facilities. We conducted the study at ‘Restwood’ (pseudonym) nursing home because the Director of Care engaged actively in the study and TREC data showed that this site differed on some areas from other nursing homes in the province. The aims of the case study were two-fold: to gain a better understanding of how frontline staff engage with the research process, and to gain a better understanding of how to share more detailed research results with management.

**Methods:**

We developed an Expanded Feedback Report for use during this study. In it, we presented survey results that compared Restwood to the best performing site on all variables and participating sites in the province. Data were collected regarding the Expanded Feedback Report through interviews with management. Data from staff were collected through interviews and observation. We used content analysis to derive themes to describe key aspects related to the study aims.

**Results:**

We observed the importance of understanding organizational routines and the impact of key events in the facility’s environment. We gleaned additional information that validated findings from prior feedback mechanisms within TREC. Another predominant theme was the sense that the opportunity to engage in a research process was reaffirming for staff (particularly healthcare aides)—what they did and said mattered, and TREC provided a means of having one’s voice heard. We gained valuable insight from the Director of Care about how to structure and format more detailed findings to assist with interpretation and use of results.

**Conclusions:**

Four themes emerged regarding staff engagement with the research process: sharing feedback reports from the TREC study; the meaning of TREC to staff; understanding organizational context; and using the study feedback for improvement at Restwood. This study has lessons for researchers on how to share research results with study participants, including management.

## Background

The field of implementation science is primarily focused on synthesizing a body of research evidence and then identifying strategies for increasing the use of this evidence in practice. Implementing evidence into practice is a complex process that may be influenced by factors such as the nature of the evidence, the context of the setting, and the facilitation processes used in the implementation process [1]. While individualized feedback has been shown to be effective in facilitating clinicians’ uptake of research knowledge [2], little is known about how to provide research findings from individuals back to organizations participating in research studies, with the aim of increasing local application. Research on implementation and feedback strategies is particularly lacking in long-term care settings. Within the fields of quality improvement and patient safety in acute care settings, data feedback mechanisms are widely used to report local data [3,4]. Research suggests that the effectiveness of data feedback depends on the quality, validity, and timeliness of the feedback, credibility and content of information [3], available dissemination channels [3], and the organizational context in which it is implemented [4]. Feedback itself is a complex interaction between format, focus, and recipient [5], and methods of providing feedback may affect the readiness of providers to engage with it [6]. The specific aims of the case study were two-fold: first, to gain a better understanding of how frontline staff engage with the research process, and second, to gain a better understanding of how to share more detailed research results with management.

## Methods

### Design

This was an intrinsic case study in which the primary focal point is the particular situation and its complexities [7]. This is in contrast to an instrumental case study that seeks to understand a particular question or process through examining that question in a particular place(s). As envisioned by Stake [7], although there is a high-level question or objective for writing the case, the specific questions emerge or are refined as the study progresses. In this study, the general goal was to increase understanding of how involvement with a program of research influences staff at a nursing home, and how it may ultimately affect resident and staff outcomes. Case study research can contribute to a more detailed understanding of how to improve care in a specific setting [8]. Ethics approval was received from the academic institution, and administrative approval was received from the facility in the spring of 2010. Data for the case study were collected through face-to-face individual semi-structured interviews and non-participant observation. Administrative support was provided by the Director of Care (DOC) and written consent was obtained from those interviewed.

### The TREC research program

The specific case study at Restwood (pseudonym) occurred as a result of it being part of a large program of research. Translating Research in Elder Care (TREC) is a multi-method five- year (2007 to 2012) program of research. In TREC we are exploring the factors that influence the use of best practices (use of research) by staff providing care in residential long-term care facilities (nursing homes) in the three Canadian Prairie Provinces (Alberta, Saskatchewan, Manitoba) [9]. We are also exploring how organizational context and the use of best practices influence staff, resident, and system outcomes [10]. Survey data were collected twice, one year apart (2008 to 2009, 2009 to 2010) from staff (healthcare aides—HCAs, professional staff, managers) working in 36 nursing homes. The Alberta Context Tool [11] was used to assess their perceptions of the organizational context and knowledge use.

As part of the TREC study, we evaluated three feedback mechanisms that focused on: post survey feedback to participating HCAs [12]; facility annual reports (FARs) to site administrators [13]; and an Expanded Feedback Report (this was one of two general aims of this case study). The purpose of the three feedback projects was to share research results with study participants in a way that would add value to their practice. In the first project, feedback posters were shared with HCAs via on-site meetings in a sub-sample of participating facilities. The report included facility-specific summary results along with comparative data, reporting the average provincial results yielded from the TREC survey. The poster included descriptive results about four variables (years staff have worked on site; percent of HCAs with formal certificate; job satisfaction; and percent of staff with time to do something extra for residents) [12]. In the second project, a FAR was mailed to each participating site’s facility administrator approximately six months after the second wave of TREC survey data collection. The four-page report was specific to each individual facility and included results from years one and two of the TREC survey data from the HCA group, which was compared graphically with the combined results for the respective province. Data on four concepts were included: workplace culture; feedback processes; job satisfaction; and staff burnout. With the exception of job satisfaction, these variables were scores comprised of several items in a scale [13].

We had arranged a site visit to meet with the DOC at Restwood nursing home because their data on the FAR differed significantly from other nursing homes in the province, and the DOC had expressed an interest in receiving more detailed results in light of these findings. The DOC engaged actively in the TREC study, and it was felt that this facility would provide a rich environment in which to observe activities relevant to TREC research. In this paper, we report findings from this third feedback mechanism project—an Expanded Feedback Report. This case study also includes results informing the second aim of the study—that of gaining a better understanding of how staff engage with the research process. This is one of a series of three papers reporting on the TREC feedback mechanism projects [12,13].

### Setting for case study

The study was conducted in Restwood, one of 36 facilities participating in the TREC study. Restwood is a nursing home in an urban location with 60 residents who reside in three ‘wings’ of the facility. The average age of residents is over 80 years, and many have moderate to severe dementia, Parkinson’s disease, schizophrenia, or anxiety disorders. Restwood is owned by a non-profit organization, and has adopted a person-oriented approach to care. A person-oriented approach is a general term for a philosophy of care that embraces dimensions such as treating each person as an individual, ensuring choice, and social inclusion [14-17]. The TREC study activities that occurred in Restwood are typical of those used in all the TREC study facilities. Table 1 provides key events occurring in Restwood with respect to the TREC research [9,10,18]. 

**Table 1:** TREC events in Restwood prior to the case study

**Date**	**Event**	**TREC personnel Involved**	**Restwood personnel involved**
Spring 2008	Advance notice regarding the survey	Letter from Principal Investigator for province	Facility manager
2008-2009	Survey 1	1 Research Assistant	15 staff from site
		1 Project Manager	
2009-2010	Survey 2	1 Research Assistant	16 staff from site
		1 Project Manager	
Concurrently	Resident Assessment Instrument—Minimum Data Set (RAI-MDS) 2.0 data being extracted	Data Manager	Facility staff collected RAI-MDS data for administrative purposes. It was accessed by TREC team for research purposes.
January 2010	HCA Poster and feedback session with staff	Research Assistant and Principal Investigator for province	Staff from site (not management at staff’s request)
February 2010	FAR sent to facility and completion of facility profile	Research Assistant	Facility manager
March 2010	Site visit	TREC Principal Investigator	Facility manager
April 2010	Teleconference to arrange case study and discuss expanded analysis desired	TREC Principal Investigator, Co-principal investigator, Case study consultant.	Facility manager

### Sample and recruitment

The interview participants comprised a convenience sample of direct care staff members and management (*i.e.*, DOC and Assistant DOC) who volunteered to participate in the case study. The interviewer/observer conducted an orientation visit at Restwood to explain the purpose of the case study. Staff and management were provided with an information letter which explained the study purpose, consent process (*e.g.*, confidentiality and anonymity), and the interview procedure. The DOC informed staff of times that the interviewer would be available (during all three shifts).

### Expanded feedback report

In addition to the two feedback reports that were sent to all participating TREC facilities (poster and FAR), one additional report was developed for Restwood specifically for use during this case study. This occurred because the DOC had previously expressed an interest in having additional analyses completed regarding the Restwood facility. After consultation between the principal investigator of TREC and the facility DOC, several variables from the TREC survey were selected for inclusion in the Expanded Feedback Report. Many of these variables were part of the Alberta Context Tool (leadership, culture, evaluation (*i.e.*, feedback processes), organizational slack (staffing, space, time), formal and informal interactions, structural and electronic resources, social capital) and the rest described various attributes of the staff (physical and mental health status, research use, job and career satisfaction, staff burnout, aggression from residents, adequate knowledge, and job orientation) [10,11]. The Expanded Feedback Report presented survey results that compared Restwood to the best performing site (in all three provinces) for each survey item as well as a comparison between Restwood and all other sites in the same province, and internally between Restwood’s year one and year two TREC results. The expanded report was a 14-page document that included a table of contents, a table of mean scores for each derived scale, coloured bar charts to show comparisons, some written text indicating statistical significance of findings, and quadrant type graphs to depict inter-relationships among variables.

### Data collection

Data were collected during a three-day period in August 2010. At that time it had been six months since the TREC survey results had been shared with staff and management at Restwood.

### Interviews with staff

Semi-structured interviews were conducted in person on site at Restwood, aimed at understanding how work is organized and done at the facility and, in particular, how changes to the way things are done in the facility come about. Examples of interview questions were: In the past year, can you think of an issue or problem that has come up in this facility? What would you say was the biggest change in how you did things this past year? and Do you recall seeing the TREC HCA report? If so, what has the report from the TREC study meant to you?

### Interview with the DOC

The Expanded Feedback Report was taken to the site and discussed with the DOC on two separate occasions during the three-day site visit. On day one, the interviewer and DOC spent two hours reviewing the report after which the research team’s data analyst was contacted to provide further information. During the process of the three days on site, the data analyst sent a document that provided greater detail and clarification on item wording. Another discussion was held on the last day of the visit (lasting one hour and including the analyst on the telephone for part of the meeting) to discuss the additional results from the analyst.

### Non-participant observation

Observations were conducted of activities in public areas within Restwood during the same three-day period and capturing all shifts. Field notes of observations were maintained.

### Data analysis

Content analysis of interview notes and field note observations was conducted to derive themes. A data record was created for each interview/meeting and one record for field notes. The records of interviews were coded initially using descriptive categories that were created because of the high level interest within the TREC program overall (*e.g.*, how TREC influenced staff) or that emerged from the interviews (*e.g.*, the energy created as a result of an artist in residence). One strong theme that emerged was the resident focus that staff consistently reiterated in the interviews. Subsequent analysis evolved and these descriptive themes emerged into interpretive themes (*e.g.*, the importance of organizational routines in using new information on best practices). Field notes from observations augmented the analysis by placing interview responses in context, providing a better understanding of the setting (*e.g.*, observations of the ‘leftover evidence’ of the artist-in-residence confirmed staff comments; observing change of shift reporting process helped illuminate the importance of organizational routines).

## Results

Interviews were conducted with a total of 13 staff members—two managers, three licensed practical nurses, and eight HCAs. Because there were two distinct lines of inquiry, results are presented separately for the components.

### Expanded feedback report

The Expanded Feedback Report was developed in hopes of providing additional information and/or support to a facility that had expressed considerable interest in understanding more about the research that had been done and using it to improve care at the facility (Restwood).

The DOC indicated that the variable ‘evaluation’ (feedback processes) that had been reported in the FAR several months earlier was not as helpful as it could be because labels did not provide any intuitive guidance about what a low (or high) score meant. The scores on individual items, as opposed to derived variables, were seen as more meaningful in some cases. Two additional variables not included in the FAR, ‘Formal Interactions’ and ‘Informal Interactions,’ were included in the expanded analysis, and the DOC commented that it was necessary to distinguish these two variables from ‘Informal Data Review’ and ‘Formal Data Review’ that were reported as part of Feedback Processes on the FAR. Hence, using unique and descriptive variable labels is helpful.

**Figure 1:**
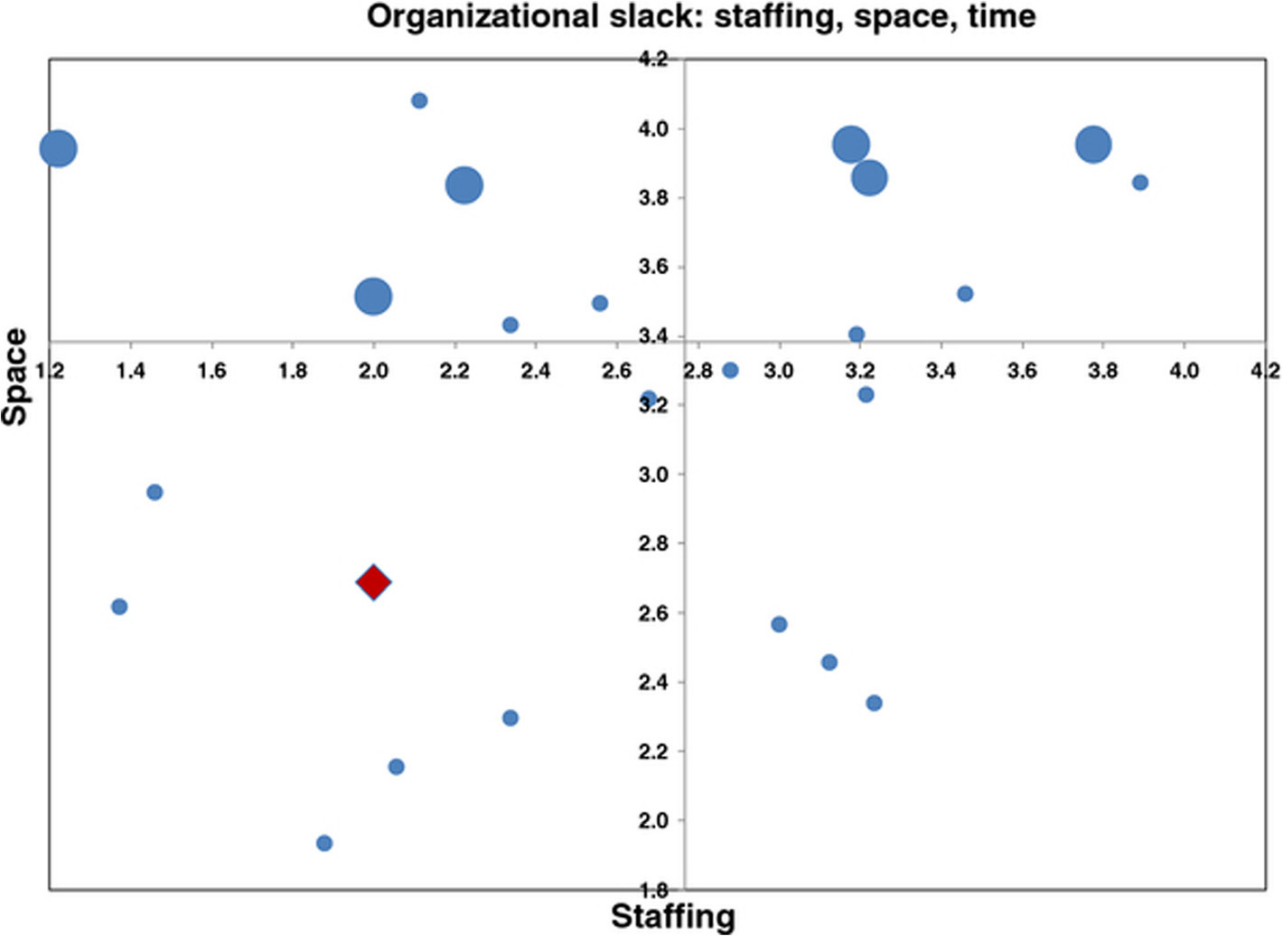
**Example of a quadrant graph showing organizational slack (staffing, space, time) results from the Expanded Feedback Report.** This is fictional data for illustration purposes. Large red diamond indicates Site A. Small dots indicate scoring below the median on Time. Large dots indicate scoring above the median on Time.

Overall, the DOC found the FAR presentation of comparative results via bar graphs of the variables understandable and helpful. However, the DOC commented that the meaning of quadrant type graphs in the Expanded Feedback Report, which depicted inter-relationships among variables, was difficult to grasp. These graphs depicted three variables in a two-dimensional space. Two variables were plotted on the horizontal and vertical axis, respectively, and the third variable was incorporated through varying sizes of dots (large if above the median on the third variable and small if below; Figure 1). In addition, the dot that reflected Restwood was shown as a large red diamond on the quadrant graphs. Hence, there were three dimensions that varied (color, shape and size) and it was not clear to the DOC which, if any, of these dimensions were meaningful when trying to compare Restwood to the other facilities. The ability to quickly glance at and get an ‘impression’ from the size and scatter of the dots (above or below the median) showing mean scores on the graph was appealing, but it was difficult to interpret with confidence the key message from these quadrant graphs.

The organization of the results in the Expanded Feedback Report was not ideal from the DOC’s point of view. In one section, the report provided scores on all variables (*e.g.*, burnout, culture, evaluation) for Restwood and compared them to the scores from the best performing facility, and in the next section of the report provided scores on all variables and compared them to the mean scores on all facilities in the province. The DOC was primarily interested in examining how the Restwood score on each variable (*e.g.*, burnout) compared to the best performing facility, then to all facilities in the province, and finally to Restwood over time. The DOC would have preferred to have the three comparisons (Restwood to best performing facility; Restwood to provincial mean; Restwood compared to itself a year earlier) presented for each variable rather than presenting the comparisons for all the variables to one comparator (*e.g.*, best performing facility). The dimension of interest was the substantive variable itself (*e.g.*, burnout) rather than the other comparison group (*e.g.*, provincial mean).

### Staff engagement with the research process

Four themes emerged from the data: sharing feedback reports from the TREC study; the meaning of TREC to staff; understanding organizational context; and using the study feedback for improvement at Restwood. Each theme is described below.

### Theme one: Sharing feedback reports from the TREC study

The TREC study feedback reports had been shared with staff (poster) and management (FAR) at the facility prior to the case study [12,13]. According to staff interviewed for the case study, the FAR had not been widely shared with staff. The FAR was seen as very helpful and informative by the DOC and most of the results resonated with the experiences of management in the facility (*e.g.*, exhaustion score from Maslach Burnout Inventory). The DOC indicated that the language was quite clear on the FAR with the exception of ‘Feedback Processes.’ It was only after the exact detailed wording of items used to capture feedback processes was shared with the DOC that the DOC understood what the Feedback Processes bar graph meant.

Two back-to-back feedback sessions were previously held with frontline staff to present and discuss the poster that showed selected findings from the HCA survey [12]. The poster was then left in the facility (on the table or posted in the staff room) for all staff to see it. However, only one-half of respondents interviewed during the case study reported ever having seen the poster. Most of those who had seen it did not recall discussing it with anyone. Although specific knowledge of TREC results was not high, most staff members were aware of ‘TREC’ (calling it by name) and greeted the interviewer with obvious knowledge of why she was there. The DOC was engaged in TREC at a more concrete and focused level than were staff (as illustrated above in discussion of the Expanded Feedback Report).

### Theme two: The meaning of TREC to staff

Three themes emerged from staff perceptions of the TREC study. First, TREC provided an avenue through which to express or demonstrate hope and a desire for change and a better future at the facility. As one staff member stated: ‘If we’re not given an opportunity to talk, nothing will ever change.’ Another staff member indicated: ‘I appreciate this research so much…at a very minimum it is addressing that there are issues in long term care…which is the first step. We need buy-in from the community.’

**Figure 2:**
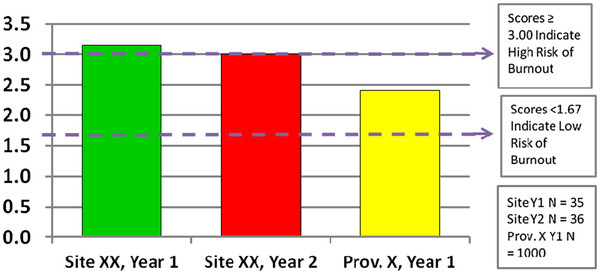
**Example of burnout results from the facility annual report.** This is fictional data for illustration purposes. Staff burnout is measured by a complex scale called the Maslach Burnout Inventory (MBI). The MBI measures three components of burnout, Exhaustion, Cynicism and Efficacy (feelings of competence and successful achievement in one’s work). The MBI consists of nine items each asking staff to describe their feelings on a 7-point scale ranging from ‘never’ having those feeling to having those feelings ‘daily.’ Example items for the three components are; Exhaustion ‘I feel burned out from my work,’ Cynicism ‘I have become more cynical’ and Efficacy ‘I am good at my work.’ The graph shows the average Exhaustion scores at your facility for years one and two compared to the provincial average. Using the high risk for burnout cut-off value (≥3.00) to identify persons at risk of burnout, the results indicate that 25% of staff scored a mean value ≥3.00 for the Exhaustion items for year one and 22% for year two. Provincially, 20% of healthcare aides scored ≥3.00 for Exhaustion.

Second, seeing results of the survey (even if for the very first time during this interview) provided a fresh lens through which to think about the current situation at Restwood. One staff member stated that the ‘exhaustion (burnout) finding for our facility is frightening’ (Figure 2). Staff were making connections in new ways. For example, looking further at the fact that the TREC study is interested in exploring the variable ‘organizational slack,’ another staff member highlighted the connection between slack and the exhaustion results (which were high for this facility).

A third and dominant theme, particularly as reflected by HCAs, was that of feeling undervalued and not listened to as staff members, and TREC provided a means of having one’s voice heard. For example, one respondent started energetically speaking even before signing the consent form about ‘being glad that someone was talking to them as they spend all their time with the residents.’ Another comment (from someone who reported having seen the HCA poster on the table) was ‘I was interested that someone is taking an interest in what is happening in our facility. To talk about the different issues that there are… it seems you work and no one pays attention.’ The fact that someone external was ‘listening’ or ‘paying attention’ to what was happening was hopeful to staff.

### Theme three: Understanding organizational context

The importance of understanding organizational routines and the impact of corporate restructuring were key themes emerging from the case study. Even equipped with the results of the TREC survey, which identifies potential areas where improvement efforts could be targeted, it is necessary to understand more about the ‘ground’ or ‘base situation’ that exists in the facility in order to strategize about how best to initiate change. The following outlines some observations from the case study that partially characterize the current situation at Restwood.

### Staffing profiles

HCAs were the vast majority of staff caring for residents compared to licensed nursing staff. Nursing staff were designated ‘in charge’ but often had less exposure (from a time point of view) to residents or to the facility operations generally than did HCAs. There were no full-time registered nurses in the facility. Therefore, some of the licensed practical nurses worked in various roles, sometimes in direct resident care and sometimes in the charge role.

### Communication routines

Regular staff meetings were held, although the frequency and attendance at these meetings was not reported consistently by all respondents. There were (at least) two streams of communication—one that related specifically to residents and another that related to other issues (policies, organizational changes). Verbal updates supplemented by brief written notes were done at shift change. Only one respondent mentioned communication books as a mechanism whereby information was shared.

### Organizational change

The ownership of Restwood had recently changed and the effects were evident from comments made by staff during the interviews. The new ownership brought with it a high-level commitment to a particular person-oriented approach. While the new ownership was not the only prevailing influence, it contributed in a major way to some observations; for example, staff attributed almost all changes that occurred in the facility (often described in a negative way as ‘rules’) to the person-oriented approach philosophy. One staff member stated: ‘POA [person-oriented approach] is not a new philosophy. But now that it’s been thrown down here with directive ‘we’re doing POA now’; now you have to do it; there’s a hard edge to it.’

However, some aspects of the person-oriented approach were deemed positive, such as a more homelike environment in the dining room. While the Restwood facility has been in existence for many years, the facility was rarely mentioned by name in interviews. There was very little evidence of ‘pride of identity’ with the Restwood facility (despite notable commitment to the well-being of residents). Staff expressed caring deeply about the residents and expressed an awareness that meaningful engagement with others makes a big difference in the quality of life for residents.

### The energy of art

There was a summer art project lead by a young ‘artist-in-residence’ that had recently occurred in Restwood. This artist was in Restwood for several weeks, and there were many paintings on the walls in the dining room and hallways. These had all been painted, framed, and hung during the recent project. A small room off the larger activity room was used for the art project. At the time of the case study, a large ‘Art Room’ sign in hand-printed letters still hung, and tables were situated around the periphery of the room with various art supplies visible. Every HCA interviewed talked about this art project positively and with a high degree of energy:

…we had a summer student absolutely phenomenal.. she brought more joy to the lives of residents than our recreation program.

A lady who lives here had never been given the opportunity to paint. This was the first time in her life… her artwork is amazing… she shakes. She can hardly hold a cup… but somehow she paints a beautiful picture… to come for one summer in a matter of three or four months… it doesn’t take much.. If you come into a facility like this and introduce something like this… it is going to be successful. Don’t know why people are afraid to introduce programs… these people need something… if it’s more interesting than sitting in a chair, it will fly…

Staff had a strong commitment to addressing the needs of the residents as illustrated through their reaction to the art project. Not only did this art experience generate much energy among the staff, the staff commented on how happy they were when the residents were happy and engaged in activities.

### Theme four: Using the study feedback for improvement at Restwood

To date, only the DOC has been involved with using the results from TREC. While there were no examples of direct action taken as a result of the research results, one action—the practice of doing annual performance reviews for all staff—was ‘spurred on’ by the TREC project. Initiating annual performance reviews (which had not been done historically) had been targeted as an objective for 2009, but participation in TREC provided further impetus as attention was focused on specific aspects of importance to staff (*e.g.*, job satisfaction, receiving recognition for work done). The DOC anticipated that the research results would provide valuable information on which to base changes in the facility.

## Discussion

We used a case study approach to explore how involvement with the TREC program of research influenced management and staff at one participating facility. The opportunity to talk with employees, share some additional results from TREC with the DOC, and observe one facility also provided insight into aspects to consider when sharing feedback reports with study participants. Our study provided insight into understanding how frontline staff engaged with the research process. Study participants indicated that their involvement in the research process provided a fresh lens through which to understand current conditions at the facility. Participating in research also provided them with a means to express their desire for positive change. However, the feedback posters which were left in communal spaces were reported to be seen by only one-half of the participants in the case study, and were seldom discussed among peers. Through this study, we also gained a better understanding of how to share more detailed research results with management. Management was satisfied with the format of the Expanded Feedback Report but suggested some content clarification, such as the use of simpler graphs and charts. Four themes emerged regarding staff engagement with the research process: sharing feedback reports from the TREC study; the meaning of TREC to staff; understanding organizational context; and using the study feedback for improvement at Restwood. This study has some lessons for our team as we move our research agenda forward and for other researchers engaged in dissemination activities, particularly when study participants are the target audience. The themes that emerged are discussed next in relation to current quality improvement and data feedback literature followed by implications for researchers and further research.

### Sharing feedback reports from the TREC study

While staff and management were pleased to have received the feedback reports from the TREC team, these reports were not widely shared with others (*e.g.*, staff). One reason for this may be that staff may not be ready to engage with research or have the time. Indeed, feedback provision is often overlooked in busy, time-constrained organizations [5]. Feedback recipients must be aware of the feedback report, and they must have access to it [6]. One lesson we learned from our feedback reporting was that in addition to meeting with staff and the DOC separately, we should hold meetings with both groups present at the same time to discuss study findings and facilitate interpretation of results and communication about them. Facilitation is central to feedback provision that fosters a process of interaction [5]. An important implication for both administrators and researchers is to facilitate and encourage processes for sharing research findings, particularly findings from studies in which staff members have participated. It is important for researchers sharing study results to be aware of existing organizational routines and practices to suggest strategies to management for applying study findings. The sharing of feedback reports is also facilitated by encouraging ownership of data through shared interpretations of findings [6]. That is, providing opportunities for researchers, staff, and management to discuss the findings together can lead to shared interpretations. Reviewing the Expanded Feedback Report with the DOC clarified potential misunderstanding of the data and provided an opportunity for the DOC to comment on the Expanded Feedback Report. A facilitated approach may encourage ownership of data and provide more sustained opportunities to discuss study findings, which may help guide efforts to a more widely sharing of research results in the future.

Characteristics of the feedback report (*e.g.*, structure and format) and who will present the reports are other areas to consider for facilitating effective feedback. Reports should be produced at a high quality and should be easy to understand. The person(s) who are presenting the feedback should ideally be research team members who conducted the study [6].

### The meaning of TREC to staff

The fact that the TREC study team had involved Restwood in the larger TREC program of research was important to staff. Valuing the work of staff and paying attention to their concerns was key to their active engagement with the TREC study. This validates findings from the HCA feedback mechanism project, which found, from the research assistants’ perspective, that HCAs were grateful to the study team for providing feedback on some of the study findings [12]. The importance of involving frontline staff in thinking about or implementing organizational change has been noted in other large programs [19,20]. Indeed, involving staff in quality monitoring and empowering them to change work activities as needed enables staff to see how their work affects resident outcomes, which can lead to improved quality of care [20]. There is evidence that providing staff with comparative performance feedback is effective in producing change in provider behaviour [21,22], particularly when combined with supports from leaders such as advanced practice clinicians (*e.g.*, gerontological clinical nurse specialist) [22]. What we have learned from the case study is the importance of selecting what is used as a comparator (*e.g.*, the DOC would have preferred three comparisons presented for each variable versus presenting the comparisons for all the variables to one comparator).

To increase the relevance and utility of research findings, it is important to understand participants’ motivations for being involved in research [23]. In this study, staff desired change for the facility and the broader context of long-term care, and they perceived TREC as a means to voice their issues and concerns. This finding is consistent with Fagnan *et al.*[23] who assessed factors that motivate clinicians to participate and stay involved in research; they found that clinicians placed high value on improving quality of care and systems of care. Participating in the TREC survey and providing staff and management with the feedback reports may act as a ‘motivating trigger’ [24] for change. For example, in each of the three feedback reports provided to staff and management as part of the TREC study, benchmarking data were provided. A study by Bradley *et al.*[4] highlighted that data meaningfulness can be enhanced through benchmarking with other organizations and one’s own organization over time, which could provide the impetus for continued improvement. Another potential motivator for change is the focus of the TREC study on staff and resident outcomes with the overall goal to improve quality of care and quality of life for residents [10]. It was evident in the case study that staff cared deeply about residents’ quality of life. In addition to the physical needs or medical care residents required, staff were committed to addressing other needs of the resident (*e.g.*, social needs), which may be as important or even more important in influencing quality of life for residents and have a direct influence on staff morale.

### Understanding organizational context

Scholars have emphasized the important role that organizational context plays in designing and implementing data feedback [4-6]. For example, it is important to consider feedback provision in a broader organizational context, such as staff time to access study findings [6] and organizational structures that influence workers’ abilities to transfer information and knowledge that inform decision making [25]. Understanding the organizational context is important in strategizing about how to provide information that may help facilities use knowledge arising from TREC and other research to initiate change. The influence may come from outside as well as inside the facility. If the workplace is heavily influenced by one or more prevailing factors (*e.g.*, change in ownership) this may affect how new information is viewed or used. If there is some activity that is generating a great deal of positive energy and momentum (such as the art project), this may provide a valuable opportunity to lever actions that support a key strategic direction.

### Using the feedback for improvement at Restwood

The TREC study has had an influence in Restwood, although to date that influence has been at a point that precedes any instrumental (direct) change in the facility. That is, the TREC research has influenced managers’ thinking about making a change; a type of research use referred to as conceptual utilization [26]. Perhaps equally important, we have demonstrated to staff and management that the work they are doing is valued. While not being directly related to the TREC findings that were shared, this may create an environment that is receptive to further engagement in using research results produced. TREC activity to date is in the early stages of communicating research results that may influence a facility’s activities. The timing of feedback may influence its effectiveness [5]. This case study was conducted approximately six months after the feedback reports from the other two feedback projects were provided to staff and management, which may not have been enough time to implement changes based on the feedback received. As well, using research findings for making improvements may be influenced by what is occurring at the facility at the time the feedback is received. Mugford *et al.*[27] found that feedback of information is more likely to influence practice if the feedback is presented close to the time of decision making or if it is part of an overall strategy that targets decision-makers who have already agreed to review their practice. Other scholars [28] have reported that leaders who are open to new experiences are likely to welcome feedback and recognize the need to act on it. Staff and management seemed to welcome the engagement with TREC, and efforts made to date to present results in understandable formats have overall been well received.

Several implications for researchers can be gleaned from these case study findings. We learned collectively with staff and management how to better present results. The structure and format of knowledge products should be tailored to specific target audiences. Groups selected to provide comparative results should be carefully chosen and researchers should understand the relevance for intended audiences. It is important to use consistent labels, descriptive phrases and abbreviations and ensure that figures are easy to understand without additional interpretation. Researchers should field test products to enhance accessibility and usefulness of results. In terms of communicating study findings, we learned that understanding organizational processes and routines may facilitate the implementation of study results into practice. Align feedback, if possible, with existing organizational routines and strategies. Hold meetings with management and staff together to share the feedback and encourage communication about the study findings. Finally, the timing of feedback may influence how it is used by feedback recipients.

Overall, the study findings reflect the key elements of the PARIHS (Promoting Action on Research Implementation in Health Sciences) framework, in which it is proposed that successful implementation is dependent upon the nature of the evidence being presented, the quality of the organizational context, and the type of facilitation required to enable a successful change process [1]. Lastly, we learned that management would benefit from support in interpreting (and perhaps also then acting on) TREC results. Results shared with different stakeholders (*e.g.*, management and direct care givers) often require different formats. These findings are consistent with the framework developed by Lavis *et al.*[2] who distinguished among four research audiences (general public, service providers, managerial decision-makers, and policy decision-makers at the federal, state/provincial, and local levels). Lavis *et al.*[2] also recommended that researchers go beyond transferring reports on research projects to transferring actionable messages based on whole bodies of research knowledge.

This study has raised several questions and areas for future research. When planning dissemination of results, it is important to consider to whom the study results are disseminated, how, when, and for how long. For example, what period of time should the provision of feedback continue; *i.e.*, what should signal the end of dissemination? It is also important to consider how organizational context influences healthcare provider and management readiness to engage with and use feedback data. What also remains unclear is what the next steps are with respect to ‘using’ the information. Under what conditions do practitioners change their behaviour in response to new information [24]? Does a particular result on one variable warrant action, or is consideration of multiple variables at the same time needed? Regular reporting of key variables could be used as ‘real time’ feedback and as a quality improvement strategy.

### Limitations

There are several limitations to this case study, some of which are inherent in the decision to view this as ‘intrinsic.’ This site was unique—changes could be envisioned, as results in some areas differed significantly from comparator sites and the on-site manager expressed interest in further learning. While this made Restwood an appealing site to study, it limits the generalizability of findings. Data were collected over a short timeframe and, although the case study provides only a ‘snapshot in time,’ conducting a case study in one organization enabled a focused exploration of staff and management perspectives on their involvement in the TREC study. Though staff interviewed involved different disciplines, further research on feedback mechanisms could include other perspectives such as senior leaders or decision-makers from outside the facility (*e.g.*, health region). The approach taken downplays the potential influence of factors outside the facility.

## Conclusions

Sharing research results with staff in nursing homes is a complex undertaking. The level of interest by frontline staff is high, but the meaning of that interest may not be as directly related to using knowledge gleaned through research as might be envisioned. Rather, in the case of TREC, it could be interpreted as laying important groundwork for future involvement in research or use of research findings. Several insights were gleaned about the importance of attending to existing organizational practices when trying to introduce use of best practices. Presenting and discussing research results that actually support a manager’s efforts to learn from them to introduce change requires careful planning and attention to detail (*e.g.*, use of unique and distinct words to label variables) and grouping results in ways that make more sense to those leading the facility. Findings from this case study will inform future approaches to providing feedback to research contributors in nursing homes.

## Abbreviations

TREC: Translating Research in Elder Care; DOC: Director of Care; HCA: healthcare aide; FAR: facility annual report.

## Competing interests

The authors declare that they have no competing interests.

## Authors’ contributions

LAC drafted the manuscript. JMB conducted the data collection and analysis for the case study and prepared a final report. DGM provided feedback on the draft manuscript. CAE and PGN conceived of the study and secured funding for the study. CAE, JMB and PGN participated in the study design and coordination and provided feedback on the draft manuscript. All authors read and approved the final manuscript.

## Authors’ information

LAC is a Postdoctoral Fellow, Knowledge Utilization Studies Program, Faculty of Nursing, University of Alberta. JMB is principal of On Management Health Group, a company that consults on organizational policy and strategy in health and health research and is a collaborator with the TREC research program. PGN is Professor Emeritus, Department of Family Medicine, University of Calgary and is the co-principal investigator for project one of the TREC research program. DGM is Professor, Canadian Centre for Health and Safety in Agriculture, University of Saskatchewan. CAE is Professor, Faculty of Nursing, at the University of Alberta and is the principal investigator for the TREC research program.
